# Effects of Structural and Compositional Changes of *Nanochloropsis oceania* after Enzyme Treatment on EPA-Rich Lipids Extraction

**DOI:** 10.3390/md20030160

**Published:** 2022-02-23

**Authors:** Kangyu Zhao, Meilan Zhang, Hua Tian, Fenfen Lei, Dongping He, Jingcheng Zheng, Liwei Zhang

**Affiliations:** 1 School of Food Science and Engineering, Wuhan Polytechnic University, Wuhan 430023, China; zhaoky95@163.com (K.Z.); zml5307@163.com (M.Z.); oilfatz@163.com (H.T.); fiona_lei@126.com (F.L.); hedp123456@163.com (D.H.); 2 Grain and Oil Resources Comprehensive Exploitation and Engineering Technology Research Center of State of Administration of Grain, Wuhan 430023, China

**Keywords:** enzyme treatment, *Nannochloropsis*, microalgae, lipidomics, structural and composition alteration, cell wall, cellulase, laccase, eicosapentaenoic acid, betaine lipid

## Abstract

Improved methods for the extraction of eicosapentaenoic acid (EPA), an essential and economically important polyunsaturated fatty acid, are urgently required. However, lipid extraction rates using food-grade solvents such as ethanol are usually low. To improve the ethanol-based extraction rate, and to elucidate the relevant mechanisms, we used cellulase and laccase to treat powdered *Nannochloropsis*, one of the most promising microalgal sources of EPA. Cellulase and laccase synergistically increased lipid yields by 69.31% and lipid EPA content by 42.63%, by degrading the amorphous hemicellulose and cellulose, improving crystallinity, and promoting the release and extraction of lysodiacylglyceryltrimethylhomoserine. Scanning electron microscopy showed that cell morphology was substantially altered, with cell-wall rupture, loss of cell boundaries, and the release of intracellular substances. In conclusion, *Nannochloropsis* lipid yields may be directly linked to cell-wall hemicellulose structure, and enzymatic treatment to alter this may improve lipid yields.

## 1. Introduction

Conventional lipid-extraction methods, using food-grade solvents such as ethanol, typically produce low yields. Given the increasing market demands for lipids, better extraction methods are urgently required. This is particularly true for eicosapentaenoic acid (EPA), an essential ω-3 polyunsaturated fatty acid with many critical nutritional functions in humans. It is the main component of cell membranes, and can effectively prevent thrombosis and cardiovascular diseases, promote fetal brain development, and ameliorate depression [1]. Increasing awareness of its importance has driven a substantial increase in demand. EPA is primarily sourced from fish oil and krill. However, wild fishing and aquaculture are unable to meet increasing global nutritional needs [2], and new sustainable sources of EPA are urgently needed [3].

Microalgae are among the most promising photoautotrophic producers of EPA [4], and are considered the most important potential industrial source of EPA [5]. Microalgae, single-celled organisms that reproduce fast, occur widely in seawater and freshwater, and are highly adaptable [6]. Microalgae synthesize high value products, such as lipids, proteins, chlorophyll, and carotenoids, via photosynthesis [7]. For instance, *Nannochloropsis* sp. F&M-M24 attained 60% lipid content after nitrogen starvation [8]. The U.S. Food and Drug Administration and European new food regulations allow the use of *Nannochloropsis* for food and nutraceuticals [9]. The National Health Commission (NHC) of China has listed *N. gaditana* as a new food ingredient (Circular No. 5, 2021).

*Nannochloropsis* primarily synthesizes polar lipids, including glycolipids, phospholipids, and betaine lipids, which form the cell membrane [10]. These membrane polar lipids contain most of the EPA in *Nannochloropsis* [11,12]. Under sufficient nitrogen, *N. oceanica* glycolipids and phospholipids comprised 48.0% and 28.4% EPA, respectively [13]. Environmental changes can cause microalgae to produce neutral (mostly intracellular) lipids, rather than glycerol-based, membrane lipids, mainly in the form of triacylglycerols [14]. To obtain EPA, extraction methods must target both membrane lipids and intracellular lipids. After lipids extraction, the solid residues from *Nanochloropsis* can be utilized as animal feeds, adsorption materials for bioremediation, fermentable carbon sources to produce biofuels or other valuable products, to realize high-value utilization of *Nanochloropsis* residues and reduce EPA production cost [15,16].

Multiple methods have been used to break cell walls. Ultrasonic crushing can be used, but its uneven energy distribution can damage intracellular substances, and it has high energy requirements [17]. Further, microalgal membrane lipids are not completely released following ultrasonic crushing [18]. Chemical methods can be applied [19], but they use toxic organic solvents that can contaminate the extracted lipids. For microalgae, enzyme-driven cell-wall breakage under weakly acidic conditions is a gentler method. Using cellulase and mannanase increased lipid yield from 40.8% (for solvent-based extraction alone) to over 73% [20]. Further, microorganisms that secrete lignocellulose-degrading enzymes have been used [21].

Freshwater and marine microalgae have a typical dense and complex trilayer structure, making their cell wall three times stronger than that of plants [22,23,24]. The thin trilaminar cell wall contains little lignin, but contains the highly aliphatic algaenan [22,25], an insoluble and non-hydrolysable biopolymer comprising long-chain aliphatic hydrocarbons bound by ether cross-linking reactions [26,27,28]. These hydrocarbons are highly resistant to alkali or acid hydrolysis and water or organic-solvent solubilization [29], making lipid extraction from *Nannochloropsis* difficult. The next layer comprises cellulose and hemicellulose and is connected to plasma membrane by struts [18]. While a single lignocellulolytic enzyme alone cannot effectively break this trilayered cell-wall, the synergistic effects of laccase and other lignocellulolytic enzymes can substantially improve breakage [21,22,25]. Nonetheless, no prior studies have evaluated their use in *Nannochloropsis* lipid extraction, or their effects on *Nannochloropsis* cell wall structure and composition.

Ethanol has low toxicity and is easily removable, making it a good substitute for the chlorinated solvents typically used to extract lipids [30]. At the appropriate concentration, it destroys the membrane bilayer structure [31]. It has been suggested that ethanol weakens or destroys the cell wall during extraction, causing lipid release via pits and crevices, and via cell lysis [32]. Few studies have addressed its application in *Nannochloropsis* lipid extraction: for *N. gaditana*, ethanol was less effective than conventional solvents for lipid extraction and EPA recovery [33]. Further study of ethanol-based lipid extraction will therefore be valuable.

To address this, we used cellulase and laccase, separately and together, to treat *N. oceanica* powder, followed by ethanol-based lipid extraction. We further examine how lipid extraction alters cell wall structure, and the mechanisms whereby these enzymes promote lipid extraction.

## 2. Results

### 2.1. Nannochloropsis Enzymatic Lipid Extraction

**Figure 1:**
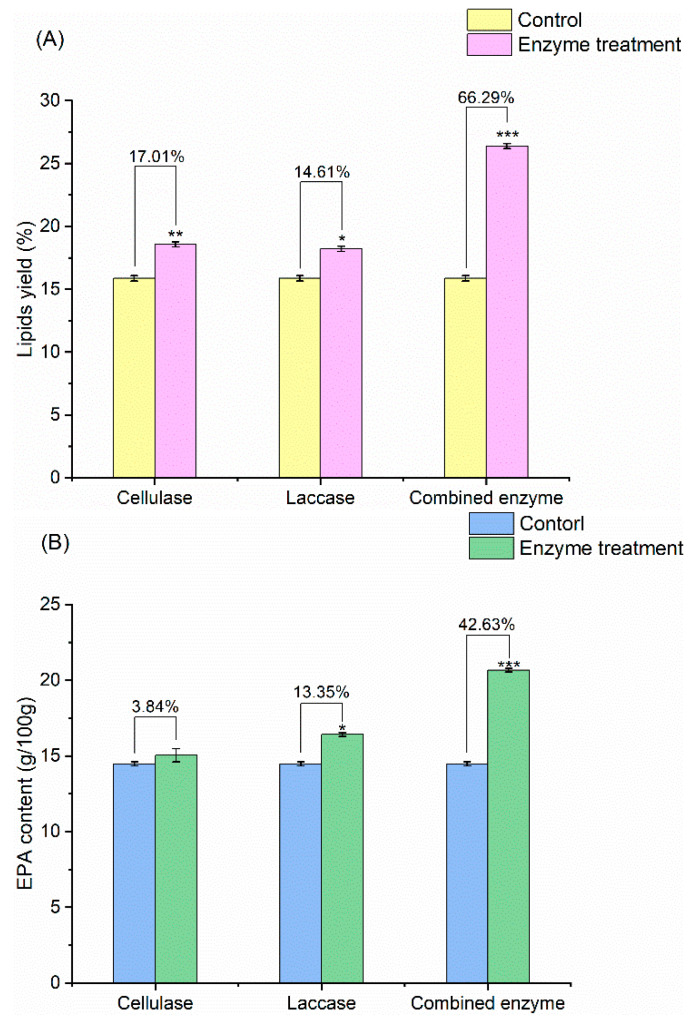
(**A**) Comparison of lipid yield of *Nannochloropsis* powder treated with different enzymes; (**B**) EPA content of microalgae powder treated with different enzymes (*: *p* < 0.05; **: *p* < 0.01; ***: *p* < 0.001).

In the current study, cellulase and laccase, separately and together, were used to treat *N. oceanica* powder, and the EPA content of lipids extracted before and after treatment was analyzed (Figure 1). Compared with the control group, cellulase and laccase increased lipid yields by 17.01% and 14.63%, respectively. The combined treatment raised lipid yield significantly (by 66.29%, Figure 1A) and EPA content by 42.63% (Figure 1B), relative to the control.

### 2.2. Lipidomics Analysis of Combined Enzyme Treatment

**Figure 2:**
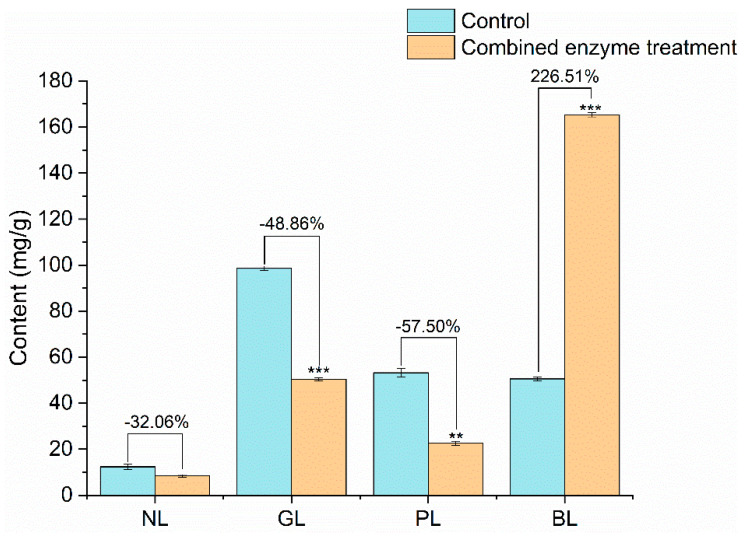
Lipid class composition of the control group and the combined enzyme group (NL- Neutral lipid; GL- Glycolipid; PL- phospholipid; BL-Betaine lipid; **: *p* < 0.01; ***: *p* < 0.001).

The lipid species identified here were consistent with those previously reported for *Nannochloropsis* [34]. Relative to the control, neutral lipid, glycolipid, and phospholipid levels were all lower after combined enzyme treatment (Figure 2). Betaine lipid levels were substantially higher (226.51%) after combined treatment.

Twelve EPA-containing lipid classes were identified: lysophosphatidylcholine (LPC), lysophosphatidylethanolamine (LPE), lysophosphatidylglycerol (LPG), phosphatidylcholine (PC), phosphatidylethanolamine (PE), phosphatidylglycerol (PG), monogalactosyldiacylglycerol (MGDG), diacylglycerol (DG), digalactosyldiacylglycerol (DGDG), diacylglyceryl trimethylhomoserine (DGTS), and lysodiacylglyceryltrimethylhomoserine (LDGTS), and triacylglycerol(TG).The EPA-containing lipids contents of the control and combination treatment groups were 108.45 mg/g and 166.21 mg/g lipid, respectively. EPA was detected primarily in the betaine lipids (DGDG, DGTS, and LDGTS). LDGTS showed the largest increase (279.96%).

### 2.3. Lignocellulose Composition

**Figure 3:**
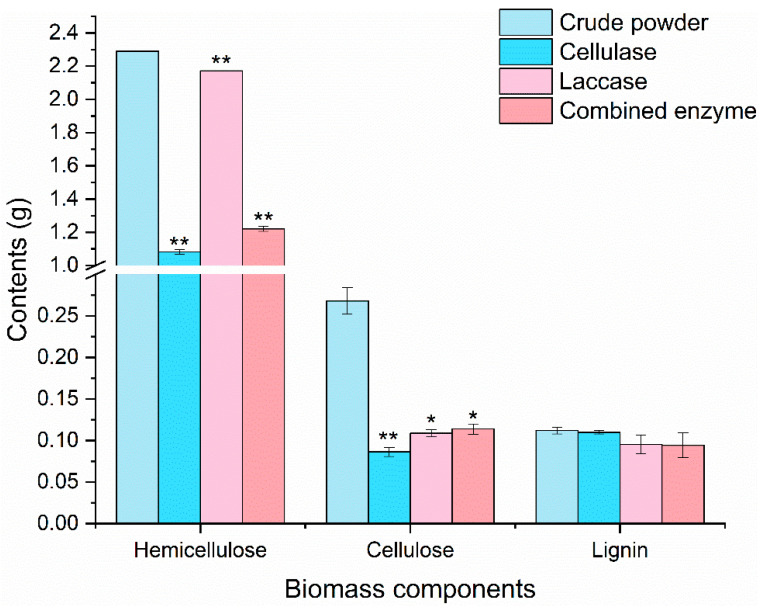
Comparison of *Nannochloropsis* lignocellulose composition before and after enzyme treatment (*: *p* < 0.05; **: *p* < 0.01).

Enzyme treatment reduced the lignocellulose content (Figure 3): combined treatment caused the largest reduction (23.40 ± 2.04%), followed by cellulase (22.60 ± 2.35%) and laccase (19.63 ± 1.68%). Combined treatment reduced cellulose by 57.50% and hemicellulose by 46.66%, that is, less effectively than cellulase. None of the treatments substantially altered lignin content. The enzyme treatment could potentially be made more effective—for instance, moderate enzymatic hydrolysis could help to improve lipid yields. Laccase did not significantly affect lignocellulose in the hydrolysed *Nannochloropsis* powder.

### 2.4. Thermogravimetric Analysis

**Figure 4:**
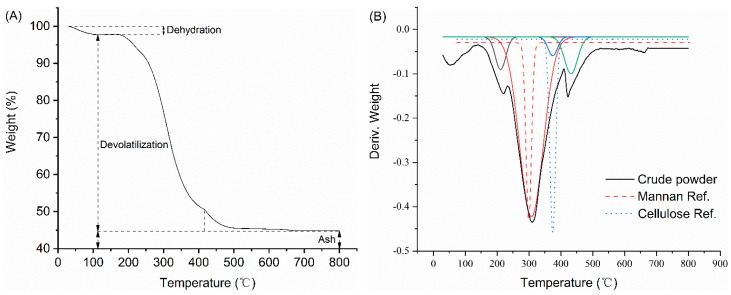

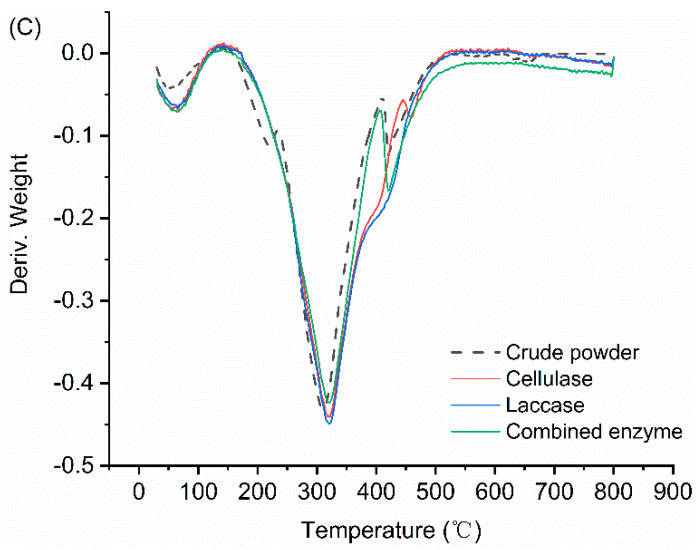
TG and DTG curves of crude microalgae powder and enzymatically treated microalgae samples. (**A**) Typical TG curve of the *Nannochloropsis* biomass; (**B**) DTG curve of crude *Nannochloropsis* powder sample with its deconvolution curves (solid lines) and cellulose/mannan standards (dashed lines); (**C**) DTG curves for crude microalgae powder sample and enzymatically treated samples.

The thermogravimetric curve (Figure 4A) indicates that *Nannochloropsis* biomass loss increased with temperature, in three stages. In the first, up to 110 °C, the main cause of biomass loss was water volatilization (dehydration). In the second, from 110 °C to 410 °C, most of the biomass loss occurred between 290 °C and 380 °C. In the third (410 °C to 800 °C), the rate of loss decreased until the biomass remained constant. In summary, >50% of the volatile substances were released at temperatures < 500 °C.

### 2.5. Fourier Transform Infrared (FT-IR) and Crystallinity

**Figure 5:**
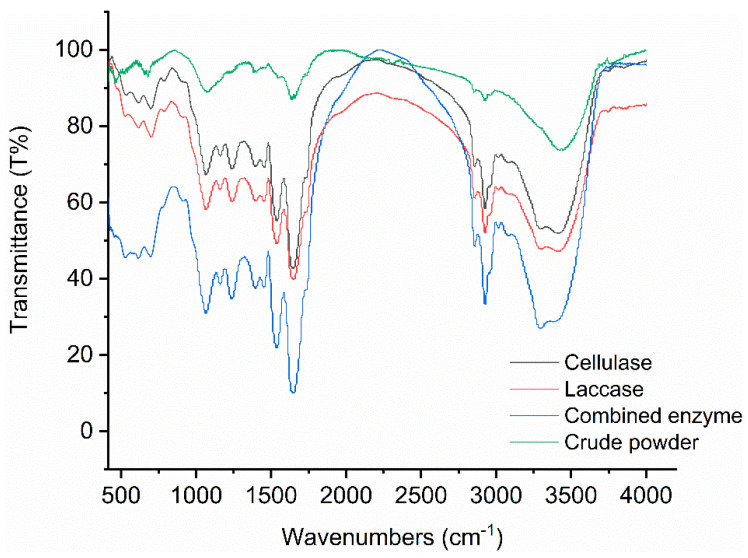
FT-IR spectrum of *Nannochloropsis* powder under enzyme treatment.

FT-IR analysis was applied for each enzyme treatment (Figure 5). The main peak areas of carbohydrates were significantly increased after enzyme treatment. The υ (C–O–C) stretching bond (around 890 cm^−1^) of cellulose and hemicellulose β-glycosidic bonds is related to enzymatic hydrolysis.

**Figure 6:**
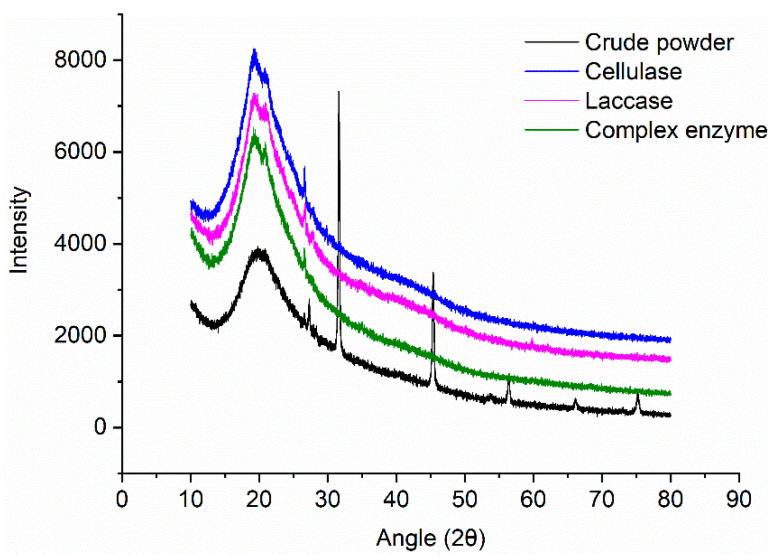
XRD spectra of enzyme-treated *Nannochloropsis* powder.

Changes in microalgal powder crystallinity following enzyme treatment were analyzed. Enzyme treatment improved crystallinity, by 51.96%, 74.00%, 74.46%, and 82.53% for control, cellulase, laccase, and combined treatment, respectively (Figure 6).

## 3. Discussion

We evaluated the effects of enzyme-driven ethanol-based lipid extraction on *N. oceanica*, focusing on cell-wall structure and lipid extract composition. The main purpose of this study was to explore the reasons for the improvement of lipid yield in *N. oceanica* by enzyme treatments.

Laccase and cellulase synergistically promoted the lipid yield and EPA content. Laccase effectively hydrolyses the algaenan in the outermost layer of the *Nannochloropsis* cell wall, enabling cellulase to hydrolyse the amorphous cellulose in the cell wall; their effect is thus synergistic [20,21]. It should be noted that we used commercial enzymes, which may contain secondary enzymes, including lipase or phospholipase. Therefore, the neutral lipid, glycolipid, and phospholipid content was lower after treatment.

Based on our lipidomics analysis, we speculate that the combined enzyme treatment increased lipid yields primarily by improving betaine lipid release. Betaine lipids, widely present in microalgae, are functional and structural polar lipids synthesized in the endoplasmic reticulum, and participating in acyl lipid–linked desaturation outside the chloroplast [35]. Here, we observed a significant increase in betaine-lipid LDGTS content, consistent with a prior *Nannochloropsis* study, in which ethanol-based lipid extraction achieved the highest LDGTS content [36]. LDGTS extracts can increase paraoxonase 1 (PON1) activity, thereby protecting macrophages and low-density lipoprotein, and possibly improving high-density lipoprotein and anti-atherogenic function [37]. In summary, our combined enzyme treatment with ethanol effectively extracted LDGTS from *Nannochloropsis* lipids. To elucidate how the enzyme treatment promotes EPA-rich lipid extraction, we analyzed its effects on cell-wall structure and composition.

We found that lignin contributed little to the lignocellulosic composition of *Nannochloropsis*. This differs somewhat from previous reports, possibly due to differences between the species studied [38,39,40]. The lost components should include components stripped from the samples after enzyme treatment, and some water-soluble substances, but not EPA or EPA-containing components. No EPA was detected in the enzyme treatment supernatant. Cellulase reduced cellulose by 67.79% and hemicellulose by 52.73%. Laccase reduced cellulose by 59.46%. This hemicellulose reduction by cellulase and cellulose reduction by laccase may be due to secondary enzymes, including other glycoside hydrolases, in the commercial enzyme products we used [21]. Further, the changes in hemicellulose structure, which were similar to those previously reported [41], might be related to lipid extraction.

We conducted thermogravimetric analysis of the *Nannochloropsis* powder after enzyme treatments, to analyze changes in structure. Based on the derivative thermogravimetry (DTG) curve, pyrolysis occurred mostly at 200–500 °C (Figure 4B). Multi-peak analysis revealed four peaks, including likely mannan and cellulose peaks, based on comparison with their respective standard curves (Figure 4B). Peaks near 200 °C were attributed to cellular and externally bound water and the release of volatile matter [42]. The peak between 400 °C and 500 °C may correspond to lipids, given that their pyrolysis occurs at higher temperatures than carbohydrates and proteins [43]. For both the cellulase and laccase treatments, the 400–500 °C peak is merged to some extent with the preceding peak (Figure 4C). This indicates overlaps, at this temperature range, in carbohydrate, protein, and lipid decomposition after enzyme treatment.

Following the enzyme treatment, most of the pyrolysis occurred at 200–500 °C, although their peaks differed. The lipid peak at 400–500 °C, following the combined treatment, is distinct from the preceding prominent peak. This may be due to our use of cellulase to treat lignocellulose, and laccase to treat the algaenan structure inside the microalgal cells [21]. These enzymes together altered the cell-walls, reducing the intermolecular hydrogen bond energy; this in turn reduces their pyrolysis temperature, and separates their peaks from the following peak.

The FT-IR spectrum was analyzed in depth. The absorption peak at 1000–1200 cm^−1^ results from C–O single-bond vibration, possibly reflecting the structural and characteristic peak of cellulose and hemicellulose, the glycosidic bond, or the ether bond between lignin and hemicellulose [44]. The absorption at 1650 cm^−1^ may reflect C=C double bond extension, while the absorption at 1250 cm^−1^ reflects C–O single bond vibration, possibly related to G-type lignin [45]. The O–H stretching near 3300 cm^−1^ and the CH_2_ stretching near 2930 cm^−1^ are characteristic cellulose absorption peaks [44]. The O–H absorption peak at 3410 cm^−1^ probably reflects the cellulose hydrogen bond [46]. As the number of hydrogen bonds in a sample increases, its structure will become looser [43]. We observed that enzymatic hydrolysis significantly increased the main peak areas. This is explained by the fact that hydrolysis degrades the cell-wall components [47]. Therefore, the cellulose, hemicellulose, and lignin characteristic groups exposed, and the numbers of hydrogen bonds detected, may be associated with the looseness of the samples. There was a strong band near 1740 cm^−1^, caused by υ (C=O) ester group stretching. This band may be derived from lipids and algaenan.

Crystallinity was related to lipid yield. We speculate that degradation of the amorphous components (part of the hemicellulose and amorphous cellulose) helps to increase the lipid yield. This is consistent with the changes in biomass composition following treatment, and with the reported finding that hemicellulose and amorphous cellulose component degradation increases *Nannochloropsis* powder crystallinity [20].

**Figure 7:**
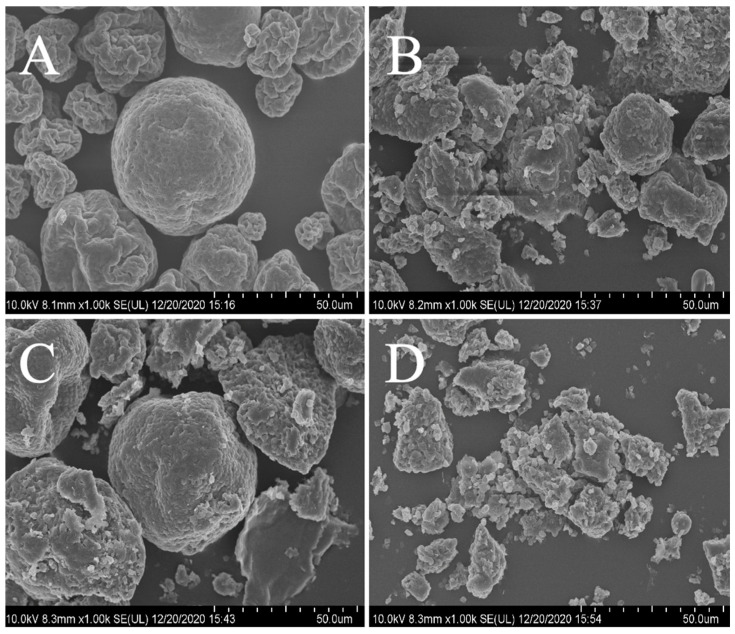
SEM images of different microalgal powder samples. ((**A**), crude powder samples; (**B**), cellulase-treated microalgal powder samples; (**C**), microalgal powder samples treated with laccase; (**D**), microalgae powder samples treated with combined enzymes).

Scanning electron microscopy revealed that cellulase alone only slightly damaged the *Nannochloropsis* cells, and laccase hardly damaged them, causing only superficial pits to appear (Figure 7).

Synergistically, they considerably altered cell morphology and caused extensive cell damage, breaking the cell wall, destroying the cell boundary, and causing the release of cell-surface and cell-content materials [20]. Based on these findings, combined enzyme treatment of *N. oceanica* may be an appropriate high-yield method to extract EPA-rich lipids for use as functional food ingredients or in nutraceuticals and pharmaceuticals. An EPA-rich diet may regulate human immunity [48], inhibit tumor-cell growth and metastasis [49], and reduce human triglyceride levels, thus reducing the risk of atherosclerosis [50].

Further, we found that combined enzyme treatment can facilitate the release of some pharmacologically active functional lipids, such as LDGTS and DGDG. However, this remains an under-explored research area. Further research is therefore required to characterize *N. oceanica* microalgae, its membrane lipids, and the lipid-concomitant compounds of each organelle, and to elucidate lipid separation by comparing with traditional method (e.g., Folch method) and the relationship between structure and pharmacologically-active substance extraction.

## 4. Materials and Methods

### 4.1. Materials and Reagents

The *N. oceanica* powder was purchased from Yantai Hairong Microalgae Cultivation Corporation Limited (Yantai, China). Cellulase from *Trichoderma viride* (50000 U/g, CAS No. 9012-54-8) and laccase from *Trametes versicolor* (120 U/g, CAS No. 80498-15-3) were purchased from Shanghai Yuanye Bio-Technology Corporation Limited (Shanghai, China). All the chemicals and solvents used were of analytical grade, unless otherwise mentioned. Fatty acid methyl esters were obtained from Sigma-Aldrich (Darmstadt, Germany). Isopropanol, methanol, acetonitrile and n-hexane were obtained from Merck KGaA (Darmstadt, Germany; all high-performance liquid chromatography (HPLC) grade). Mixtures of deuterated lipid internal standards were obtained from Avanti Polar Lipids (Alabaster, AL, USA).

### 4.2. Enzymatic Treatment

*Nannochloropsis* microalgae powder was mixed with citric acid/sodium citrate buffer (pH 5.0; 1:5 *w/v*) and stirred gently. Cellulase and laccase were then added to the mixture separately, as the single-enzyme treatments, or together, for the combination treatment. The final concentrations of the enzymes were the same in each group (cellulase 500 U/mL, laccase 0.24 U/mL). The enzyme treatments were conducted under the same conditions. The enzymatic hydrolysis temperature was 50 °C, and it was conducted for 24 h. The enzymatic hydrolysis suspension was then centrifuged at 2325× *g* for 15 min, and the lower sediment was taken, dried, and analyzed.

### 4.3. Lipid Extraction

Ethanol extraction method: We added 90% (*v/v*) ethanol–water solution to the microalgal powder (1:15 *w/v*), stirred it gently, and placed it at the extraction temperature of 65 °C for 3 h. The suspension was then centrifuged at 3488× *g* for 15 min. The supernatant was taken and steamed at 80 °C for 30 min to obtain the lipids. Meanwhile, Folch method was applied as comparison [51]. Lipid yield was calculated as follows:

$$\mathrm{Lipid}\;\mathrm{yield}\;\left(\%\right)=\frac{wt\;\mathrm{of}\;\text{solvent-free }\mathrm{extract}}{wt\;\mathrm{of}\;\mathrm{microalgae}\;\mathrm{powder}}\;\times \;100\%$$

### 4.4. Lipid EPA Content

#### 4.4.1. Fatty Acid Methyl Ester Preparation

A modified method based on the literature was used [52]. First, 200 mg of lipid sample was placed in a 10 mL glass tube with 2 mL of KOH-CH_3_OH (0.5 mol/L); the mixture was then shaken at 60 °C for 30 min. Then, 2 mL of BF_3_-CH_3_OH (50 wt%) was added to the mixture, which was bathed at 60 °C while being shaken for 3 min. After being cooled to 25 °C, saturated NaCl solution and chromatographic grade n-hexane (2 mL each) were added to the mixture and shaken. After being left to stand, the supernatant was taken and passed through a 0.25 μm filter membrane to be analyzed by gas chromatography (GC).

#### 4.4.2. Gas Chromatography

The injector and detector were both maintained at 250 °C. An Agilent HP-88 capillary column (100 m × 25 μm; 0.25 μm) was used. The heating program was as follows: 140 °C for 2 min, 3 °C/min increase to 185 °C, hold for 5 min, 3 °C/min increase to 200 °C, hold for 3 min, 2.5 °C/min increase to 225 °C, and hold for 5 min. The injection volume was 1.0 μL, and the split ratio was 1:40.

The lipid EPA content was determined via the external standard method, according to the Chinese National Standard GB 5009.168-2016.

### 4.5. Lipidomic Analysis

The lipidomics were determined based on the literature methods [53].

Samples were prepared as follows: 10.0 mg of sample lipid was dissolved in 1 mL of isopropanol, then vortexed for 30 min to fully dissolve it. Isopropanol was then added to dilute each sample 100 times, and 10 μL of the deuterated lipid internal standard mixture was added (100 μg/mL). Each sample was then vortexed to ensure mixing, before being centrifuged at 6976× *g* for 5 min. The supernatant was then filtered through a 0.22 μm organic filter membrane for testing.

The ultra-high-performance liquid chromatography conditions were as follows: the chromatographic column used was a Phenomenex Kinete C18 (100 mm × 2.1 mm; 2.6 μm), the column temperature was 60 °C, the injection volume was 1 μL, mobile phase A was H_2_O-methanol-acetonitrile (1:1:1 *v*/*v*/*v*, containing 5 mmol/L NH_4_Ac), mobile phase B was isopropanol-acetonitrile (5:1 *v*/*v*, containing 5 mmol/L NH_4_Ac), and the flow rate was 0.4 mL/min. The gradient elution program is shown in the Table 1.

The mass spectrometry conditions were as follows: an electrospray ionization (ESI) ion source was used in positive mode. The primary mass spectrometry acquisition mass-to-nucleus ratio range was 100–1200, and an AB Sciex TripleTOF 6600 system was used, using these conditions: curtain gas, 35.000 psi; ion source gas 1, 50.000; ion source gas 2, 50.000; ion spray voltage, 5500.00 V; and temperature, 600 °C.

### 4.6. Lignocellulose Composition Analysis

Lignocellulose composition was analyzed using the Van Soest method [54].

### 4.7. Thermogravimetry (TG)

Thermal analysis, including thermogravimetry and derivative thermogravimetry, were performed on a Thermal Gravimetric Analyzer (METTLER TGA/DSC/1100SF, Switzerland). Testing conditions were based on the literature [20]: temperature range, 30–800 °C; nitrogen protective airflow rate, 25 mL/min; and heating rate, 10 °C/min.

### 4.8. Fourier Transform Infrared (FT-IR)

We mixed and ground the microalgal sample (1 mg) and potassium bromide (100 mg). The evenly ground powder was then made into potassium bromide tablets for FT-IR detection (Thermo Nicolet 6700, Waltham, MA, USA). The scanning wavenumber was between 400 cm^−1^ and 4000 cm^−1^, based on the literature [55].

### 4.9. X-ray Diffraction

X-ray diffraction (Bruker D8 Advance, Berlin Germany) was used to determine crystallinity. The test conditions were as follows: 0.15406 nm, 40 kV, 40 mA, scanning range 10–80°, scanning speed 10°/min. Jade 6.0 (Materials Data, CA, USA) was used for data analysis and crystallinity calculation. The crystallinity calculation method is provided in the literature [56].

### 4.10. Scanning Electron Microscopy

Microalgal morphological changes following enzymatic treatment were analyzed using a Field Emission Scanning Electron Microscope (Hitachi SU8010, Tokyo, Japan). The preparation method of microalgae cells was referred to the literature [20].

### 4.11. Statistical Analysis

The trials were performed in triplicate. Data were analyzed using Microsoft Excel 2019 and Origin 2018. The results are presented as mean ± standard deviation. One-way ANOVA was performed in IBM SPSS 26.0 (SPSS Inc., Chicago, IL, USA). We compared means using Duncan’s multiple range tests, and differences were considered significant at *p* < 0.05.

## 5. Conclusions

The combined cellulase and laccase treatment improved *Nannochloropsis* lipid yield more effectively than single-enzyme treatment, possibly by synergistically altering cell-wall structure, and hemicellulose and cellulose contents. The enzyme treatment degraded the amorphous microalgal components, and promoted lipid extraction, mostly by promoting the release and extraction of betaine lipids, primarily LDGTS. Future research, focusing on lipidomic analysis of *Nannochloropsis*, should aim to separate and purify the lipid components or their concomitant compounds, to promote the development and utilization of this emerging resource.

## Figures and Tables

**Table 1:** Mobile phase gradient elution procedure.

Time/min	A/%	B/%	Time/min	A/%	B/%
0.5	80	20	13	2	98
1.5	60	40	13.1	80	20
3	40	60	17	80	20

## Data Availability

The data presented in this study are available on request from the corresponding author.
